# Neuropathy 10–15 years after Roux-en-Y gastric bypass for severe obesity: A community-controlled nerve conduction study

**DOI:** 10.1016/j.cnp.2024.03.002

**Published:** 2024-03-27

**Authors:** Trond Sand, Arnstein Grøtting, Martin Uglem, Nils Augestad, Gjermund Johnsen, Jorunn Sandvik

**Affiliations:** a Department of Neurology and Clinical Neurophysiology, St. Olavs Hospital, Trondheim University Hospital, Norway; b Department of Neuromedicine and Movement Science, Faculty of Medicine and Health, Norwegian University of Science and Technology, Trondheim, Norway; c Department of Surgery, Møre and Romsdal Hospital Trust, Ålesund, Norway; d Centre for Obesity Research, Clinic of Surgery, St. Olavs Hospital, Trondheim University Hospital, Norway; e Department of Clinical and Molecular Medicine, Faculty of Medicine and Health Sciences, Norwegian University of Science and Technology, Trondheim, Norway; f Norwegian National Advisory Unit on Advanced Laparoscopic Surgery, Clinic of Surgery, St. Olavs Hospital, Trondheim University Hospital, Norway

**Keywords:** Obesity (BMI), RYGB-Surgery, Nerve conduction study (NCS), Carpal tunnel syndrome (CTS), Polyneuropathy (PNP)

## Abstract

**Objective:**

We searched for long-term peripheral nerve complications 10–15 years after Roux-en-Y gastric bypass surgery (RYGB), using a comprehensive nerve conduction study (NCS) protocol.

**Methods:**

Patients (n = 175, mean age 52.0, BMI 35.2) and 86 community-controls (mean age 56.8, BMI 27.2) had NCS of one upper and lower limb. New abnormality scores from 27 polyneuropathy-relevant (PNP27s) and four carpal tunnel syndrome-relevant NCS-measures (CTS4s) were compared between groups with non-parametric statistics. Estimated prevalences were compared by 95 % confidence limits. The clinical neurophysiologist’s diagnosis was retrieved from hospital records (PNP-ncs, CTS-ncs, other).

**Results:**

Abnormality score did not differ between RYGB and control groups (PNP27s: 1.9 vs 1.7, CTS4s: 0.7 vs 0.6, p > 0.29). BMI correlated weakly with CTS4s in patients (rho = 0.19, p = 0.01), and less with PNP27s (rho = 0.12, p = 0.12). Polyneuropathy (PNP-ncs) prevalence was 12 % in patients and 8 % in controls. CTS-ncs prevalence was 21 % in patients and 10 % in controls (p = 0.04).

**Conclusions:**

NCS-based abnormality scores did not differ between patients 10–15 years after RYGB and community-recruited controls, neither for PNP nor CTS.

**Significance:**

Long-term polyneuropathic complications from RYGB have probably been avoided by modern treatment guidelines. NCS-diagnosed CTS is common in overweight RYGB patients. RYGB-patients with significant neuropathic symptoms need clinical evaluation.

## Highlights

• NCS-determined axonal PNP-risk was low 10–15 years after RYGB surgery.
• NCS-diagnosed CTS was commonly found among overweight RYGB operated patients.
• New NCS-based abnormality scores correlated weakly with BMI for CTS but not for PNP.

## 1 Introduction

Roux-en-Y gastric bypass surgery (RYGB) is a common treatment for severe obesity (Mason and Ito, 1969, Carrano et al., 2022). It has been estimated that about 5 % develop neurological complications after surgery (Abarbanel et al., 1987, Goodman, 2015) while estimates from retrospective studies may vary from 1.3 % to 17 % (Koffman et al., 2006, Thaisetthawatkul et al., 2004). Cobalamin deficiency was previously a typical cause for polyneuropathy (PNP), and regular oral or parenteral vitamin B12 supplement has been standard treatment for many years now. Thiamine deficiency is another well-known cause for subacute PNP, sometimes complicated by Wernicke’s encephalopathy, and these conditions must be treated adequately and fast to avoid permanent damage to neural tissue. (Landais, 2014, Goodman, 2015).

Obesity itself, particularly in those with comorbid prediabetes and diabetes, seem to be a risk factor for clinical PNP (Callaghan et al., 2016). However, most of their nerve conduction study (NCS) measures did not differ between patients with obesity without clinical PNP and lean community-controls. Few NCS evaluations of patients with obesity have been reported, and there has been concerns about artificial reduction of NCS-amplitudes, a common measure for axonal neuropathy, among patients with obesity without PNP (Buschbacher, 1998). In addition, no long-term follow-up on peripheral neuropathy more than ten years after gastric bypass operations seem to exist.

The present study is part of the Bariatric surgery observation study (BAROBS) on the health after previous RYGB surgery performed 10–15 years ago in three hospitals within the Central Norway Regional Health Authority (Strømmen et al., 2021, Sandvik et al., 2021). Our general aim was to study peripheral neurological complications in this population, using a comprehensive NCS protocol, since electrodiagnostic studies are validated, sensitive and specific measures of polyneuropathy (England et al., 2005) that yield important additional information in patients referred with a clinical PNP diagnosis (Ginsberg and Morren, 2020). Our working hypothesis was that NCS-based neuropathy would be more frequent among RYGB patients than controls. Our primary objective was to estimate the prevalence of objective NCS-based PNP, carpal tunnel syndrome (CTS) and other neuropathies 10–15 years after RYGB surgery. Prevalences and severity were estimated by new summed categorical abnormality scores for PNP, since combined NCS scores are sensitive and specific measures of distal symmetrical PNP (Solders et al., 1993, Dyck et al., 2011b, Dunker et al., 2022). Summed CTS-scores were also applied. We also estimated prevalences from the attending clinical neurophysiologist’s signed conclusions. Secondary objectives were to study the association between NCS, body mass index (BMI) and known disease, and to determine which parts of the peripheral large-fibre somatic nervous system were most affected, either sensory or motor nerves in the arms and legs.

## 2 Methods

### 2.1 Subjects

In total 959 patient underwent RYGB surgery from 2003 to 2009, 29 died during this period and 930 subjects living in Norway were invited to the follow up study. Five hundred and forty-six patients appeared at follow-up and were included in the main BAROBS study. Patients had to speak Norwegian language. Specific exclusion criteria were not applied. In the protocol we aimed to recruit one third of the patients for this NCS sub-study. One hundred and eighty-nine patients with previous RYGB-surgery performed either at St. Olavs Hospital in Trondheim or in Ålesund Hospital were randomly selected and invited for a neurological sub-study. Five had known CTS, two had experienced previous ulnar neuropathy at the elbow and one reported a previous peroneal palsy. By chance, no patient with known PNP-history was included. One hundred and seventy-five patients completed the NCS examination for the present study. Pre-surgery NCS were not available.

The RYGB-group was compared to a community-based community-control group from the HUNT4 study (The Trøndelag Health Study, wave 4). Two hundred and thirty-two randomly selected subjects from the Stjørdal region had been interviewed for headache and insomnia (Filosa et al., 2021), and 86 of them had accepted additional NCS examinations of one arm and one leg from February 2018 to September 2019, at St. Olavs Hospital. Three had previous CTS-treatment and one had been operated for ulnar nerve entrapment. One subject had previous PNP after drug treatment for cancer.

Informed consent was applied. BAROBS (REK (ref. nr: 2017/1828 REK sør-øst B) and HUNT4 studies (2019/804/Rek Midt) were approved by the Regional ethics committees.

### 2.2 NCS methods

NCS in both arm and leg were successfully recorded in 175 patients (72 in Trondheim and 103 in Ålesund; the RYGB-group) from September 2019 to March 2020. NCS included motor and sensory conduction velocities (MCV and SCV) and compound muscle and sensory nerve action potential (CMAP and/or SNAP) amplitudes from median, ulnar, peroneal, sural, tibial and medial plantar nerves. One patient did not have ulnar nerve motor data by mistake. Low-amplitude non-recordable SNAPs were imputed with zero µV before analysis.

Ulnar entrapment was evaluated by MCV and the proximal CMAP-amplitude along 10 cm segments across the elbow. Peroneal entrapment was evaluated by MCV and the proximal CMAP-amplitude across 10 cm segments at the fibular head. CTS was evaluated by four standard variables: distal motor latency (DML) from wrist to the thenar eminence, orthodromic SCV from digit 3 to wrist, mixed SCV from volar surface to wrist, and the digit 5 minus digit 3 SCV-difference. Four additional CTS-variables were also applied: SCV from median (lateral branches) dig 4 to wrist and the SCV-difference compared to the ulnar dig 4 (medial branches), median DML to second lumbrical and the motor DML-difference between the median segment from the wrist to second lumbrical and the ulnar segment from medial wrist to the second interosseous muscle. Hands and feet were pre-heated by packs and infrared light to measured 32–34 °C skin temperatures. Self-adherent surface electrodes were applied to the skin (pre-gelled electrodes with recording area of 9 mm × 6 mm (Alpine Biomed ApS, Skovlunde, Denmark). Stimulation was provided by fixed bipolar felt pad electrodes, and 1 Hz, 0.2 ms sqare wave pulses adjusted to supramaximal intensity for motor nerves and to either supramaximal or four times sensory thresholds for sensory nerves. Twenty F-responses were recorded, and the minimal (F-M min) F-M difference latencies were calculated for the four motor nerves.

A DeyMed EMG machine with TruTrace 7.08 software (Deymed diagnostic s.r.o, Hronov, Czech republic) was used at St.Olavs hospital. Ålesund hospital used partly the same machine (n = 31) and partly (n = 72) a Keypoint G4 machine with Keypoint Classic 5.13 software (Medtronic, Copenhagen, Denmark).

NCS-measures were scored as normal (0) or abnormal (1) compared to age and height-corrected normal values from our reference database (n = 717, 70 % women, mean age 45 years, range 13–86 years, mean height 171 cm, range 149–198 cm). Normal limits were calculated from normally transformed age and height corrected regression lines ±2 SD.

### 2.3 NCS evaluations

Twenty-seven single NCS-variables (Table S1) were selected (by clinical experience) for calculation of the summed abnormality score PNP27s. All 27 measure scores (either score 0 or 1) were summed yielding a possible range 0–27 for the PNP27s variable. PNP sub-score sums for 15 motor and 12 sensory nerves, and for 13 upper and 14 lower extremity nerve measures were also calculated. The four standard CTS variables (Table S2) were selected for a summed score for carpal tunnel compression (CTS4s; range 0–4). The four additional CTS-measures were available in the RYGB group for the CTS8s summed score. Supplementary Tables S1 and S2 show the NCS variables.

In addition, all NCS examinations were evaluated by experts, certified MD-specialists in clinical neurophysiology (TS or AG) and documented in the electronic patient journals (EPJ). Abnormality scores were not used during clinical evaluation. All EPJ-reports were retrieved and scored (NA and TS). Conclusions with wordings interpreted as either definite or probable neuropathy were pooled for the present analysis. The EPJ-conclusions were to be categorised as axonal NCS-determined polyneuropathy (PNP-ncs), NCS-determined demyelinating PNP, NCS-determined carpal tunnel syndrome (CTS-ncs), and NCS-determined ulnar entrapment, peroneal entrapment and other mononeuropathies.

### 2.4 Clinical evaluation

RYGB patients also had clinical examinations by attending physicians (four surgeons, one general practitioner and one resident) and questionnaires administered by study nurses. It was not possible to recruit certified neurologists to perform a full neurological examination, but physicians were trained by a neurologist in the neurological examination protocol. The structured neurological examination included Romberg’s test, walking balance, finger-nose and knee-heel dysmetria tests, and five sensory tests (normal or abnormal) according to the standard version of Toronto clinical neuropathy score (TCNS) protocol (Bril et al., 2009). The examination for tendon reflexes was not performed due to lack of time. Sensory system examination included vibration sense at the medial malleolus by tuning forks and sensation to light touch, cold, and pin-prick at the big toes. Six questions about foot pain, numbness, tingling, weakness, ataxia, and upper limb neuropathic symptoms were posed according to the TCNS symptom scores (absent or present). Patients also reported comorbidities like diabetes, cancer history, alcohol consumption habits and hypertension. A TCNS-based summed sensory score (possible range 0–11) was computed. A 5.07/10 g Semmes-Weinstein monofilament sensory test (SWME) was also applied under both feet (American Diabetes Association, 2018).

Community-controls did also have a structured neurological examination for PNP including the full TCNS score. Five certified specialists in clinical neurophysiology took part in the clinical evaluation of community-controls. From the clinical interviews and examinations in both groups, the physician concluded, by clinical experience, whether the patient had a definite peripheral neuropathy or not (possible answers: yes, no, do not know).

### 2.5 Statistical methods

Abnormality rates with 95 % confidence intervals for different cut-offs for summed PNP27s and CTS4s scores are reported as main outcomes and compared with chi-square tests. Prevalences for NCS-determined neuropathies recorded in the patient journal were also compared between groups. Rates within three BMI subgroups (<25, 25–35, and ≥35 kg/m^2^) were compared with chi-square tests and Cochran’s linear trend in the RYGB group. Spearman non-parametric correlation coefficients (rho) for BMI, CTS4s, and PNP27s were calculated in RYGB patients. Summed abnormality scores (PNP27s, CTS4s, and the four PNP-sub-scores) were also compared between groups with a non-parametric Mann-Whitney test. Descriptive Student’s t-tests or chi-square tests were included for background variable group differences.

For sensitivity analysis we created BMI- and sex-balanced subgroups by selecting paired participants with closely matching BMI values and sex, thus excluding RYGB subjects with BMI above 39 kg/m^2^ (the maximum BMI in controls) and BMI below 21 kg/m^2^ (the minimum value among patients). The BMI range among 108 subgroup participants was from 20.5 to 38.1 kg/m^2^. Controls (n = 54, 69 % women, age 57.0 (sd 10.4), BMI 28.7 (sd 4.4) and RYGB (n = 54, 70 % women, age 53.2 (sd 9.8), BMI 28.8 (sd 4.3).

Systat 13.1 and SPSS v28 were used for statistical analysis. A two-sided p-value < 0.05 was considered significant. Power in independent Student’s t-tests comparing 175 and 86 subjects is 80 % to detect a rather small real effect equal to 0.37 × SD (Lachin, 1981).

## 3 Results

### 3.1 Subjects

The RYGB-group was slightly younger, had slightly more women (p = 0.01) and (by study design) substantially higher BMI than community-control subjects. Present diabetes, alcohol use, cancer history and hypertension rates were similar in the two groups. A history of sciatica was reported by 30 % of our RYGB-patients and 16 % of controls (p = 0.02). RYGB patients reported more sensory symptoms, while objective sensory deficits were uncommon and only slightly more prevalent among community-controls. TCNS sensory scores were higher in RYGB patients although means were low in both groups. The attending physician’s definite clinical PNP-diagnosis yielded similar results in the two groups (6.3 % vs. 8.1 %; Table 1).

**Table 1:** Population demographics for RYGB bariatric surgery patients and population-based controls from the HUNT4-study.

	**RYGB group (n = 175)**	**Community-controls (n = 86)**
	**Mean (sd) or percent**	**Mean (sd) or percent**
Women	79 %	65 %^1^
Age (yrs)	52.0 (9.2)	56.8 (11.5)^2^
Height (cm)	168.8 (8.3)	170.2 (8.5)
Weight (kg)	100.5 (23.0)	79.0 (15.5)^2^
BMI (kg/m^2^)	35.2 (7.2)	27.2 (4.4)^2^

Time from RYGB (months)	138.3 (19.8)	–
Age at RYGB (yrs)	39.9 (9.0)	–
BMI at RYGB (kg/m^2^)	44.2 (5.7)	–
BMI-minimum (kg/m^2^)	29.4 (5.0)	–
Diabetes T2 at RYGB	16 %	–
Hypertension at RYGB	18 %	–

Diabetes T2 now	7 %	5 %
Hypertension now	22 %	22 %
B12 supplements	97 %	2 %^2^
History of sciatica	30 %	16 %^1^
Alcohol >3 days a week	2 %	5 %
Cancer-history	6 %	4 %

Physician’s definite clinical PNP diagnosis	6 %	8 %
Monofilament 10 g test abnormal	3 %	7 %
Neuropathic arm symptoms	39 %	14 %^2^
Leg pain	36 %	7 %^2^
TCNS sensory symptom score (0–6) ≥ 2	44 %	18 %^2^
TCNS sensory test score (0–5) ≥ 2	11 %	19 %
TCNS both sensory test and symptom scores ≥ 2	6 %	4 %
TCNS sensory sum score^a^ (0–11) ≥ 3	35 %	22 %^1^
TCNS sensory sum score^a^ (0–11) mean (sd)	2.2 (2.2)	1.4 (1.7) ^2^
TCNS total score (0–19)	na	2.6 (3.0)

PNP: Polyneuropathy rates based on clinical examination by medical doctors. T2: Type 2 diabetes. TCNS: Toronto clinical neuropathy scale. na: not available (achilles and patellar tendon reflexes were not performed in the RYGB group).

Descriptive statistical comparison (Student’s *t*-test or chi-square test) for background variables: ^1^p < 0.05, ^2^p < 0.005.

a sum of sensory symptom and sensory test score (possible range 0–11).

### 3.2 NCS abnormality scores

**Fig. 1:**
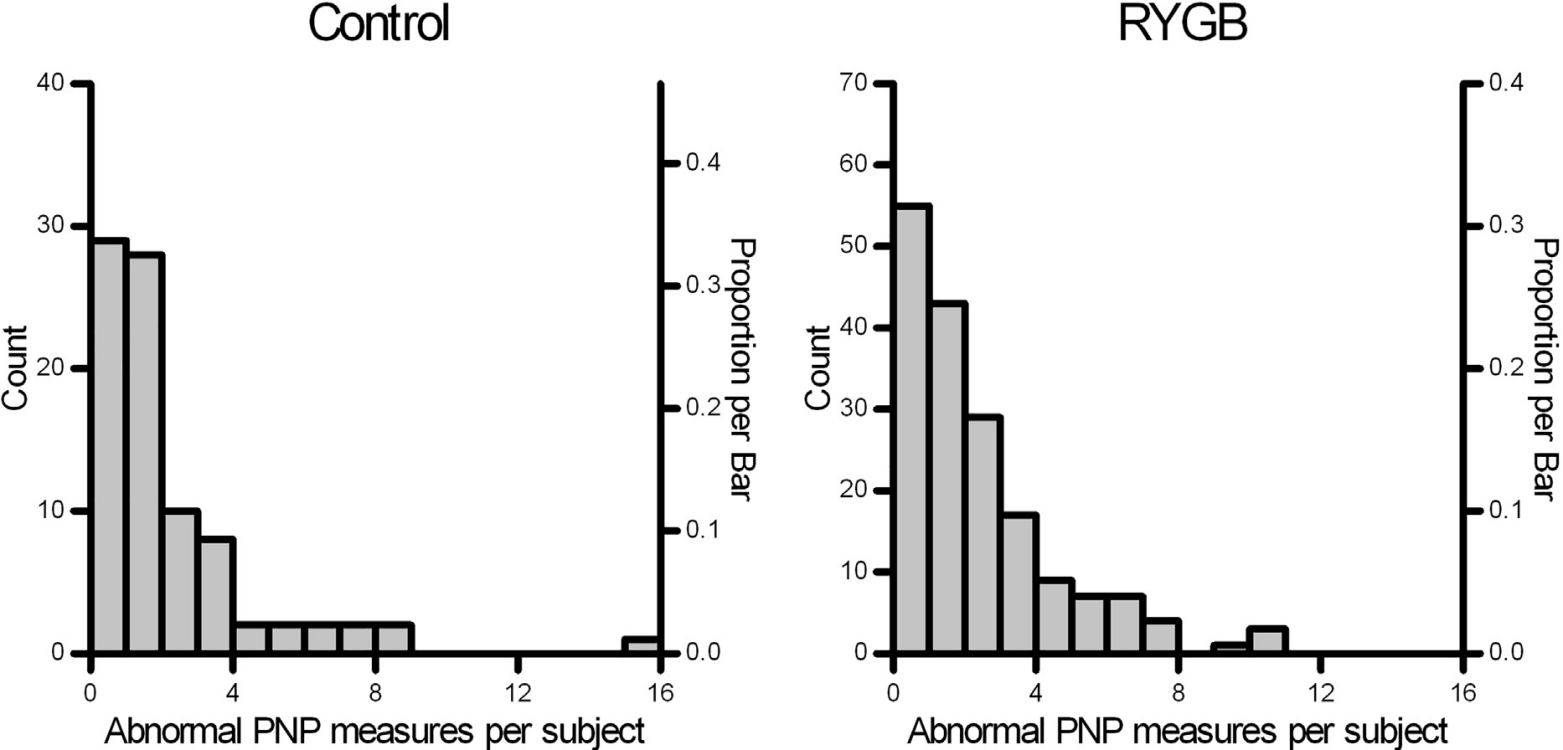
NCS abnormality-scores for polyneuropathy. Distributions show the number and proportions of abnormal NCS-measures per subject in RYGB (n = 175) and community-control (n = 86) groups, for 27 polyneuropathy-relevant measures (PNP27s). The distribution shapes are similar between groups as shown by a non-significant chi-square (df 11) = 12.5, p = 0.33).

**Fig. 2:**
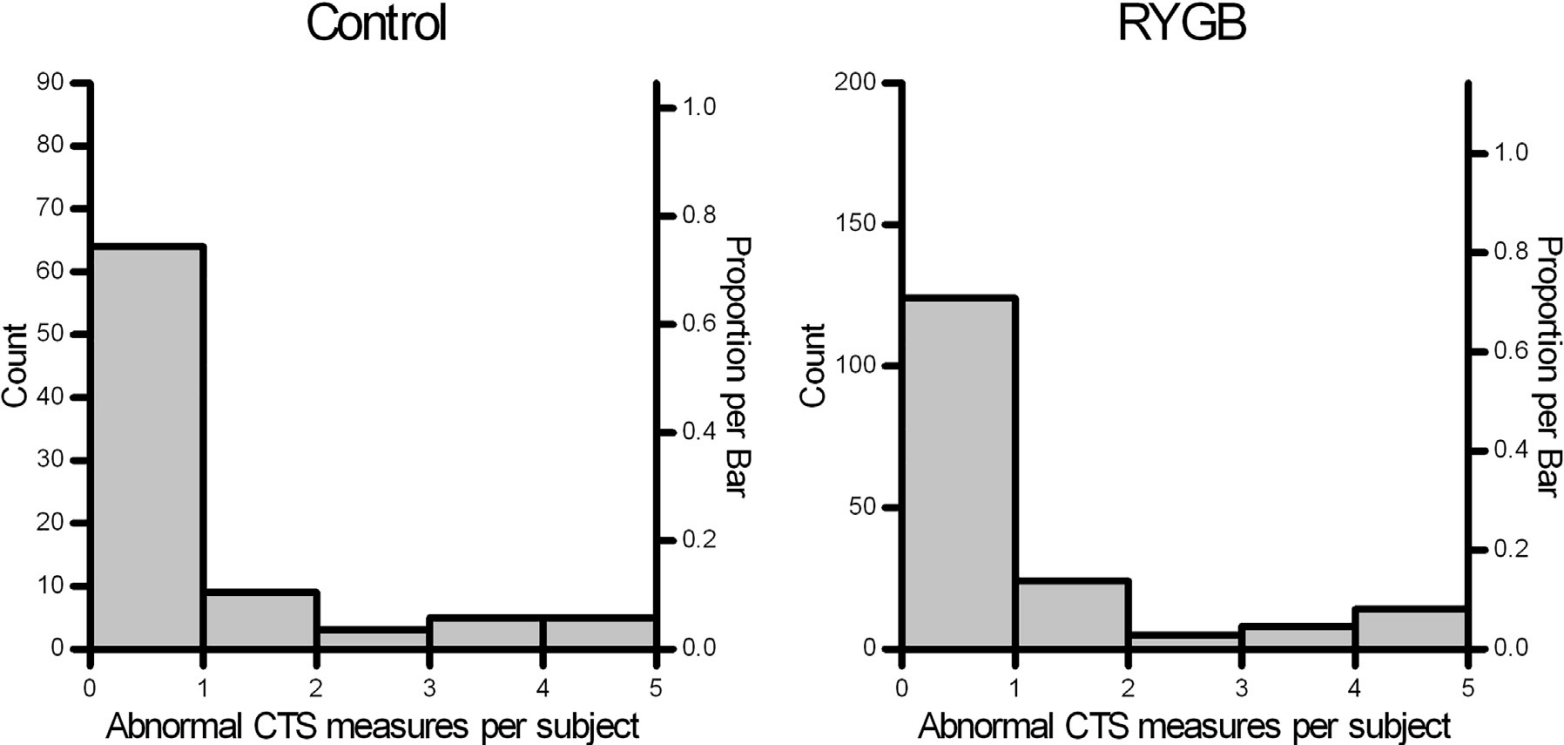
NCS abnormality-scores for carpal tunnel syndrome. Distributions show the number and proportions of abnormal NCS-measures per subject in RYGB (n = 175) and community-control groups (n = 86) for four carpal tunnel specific measures (CTS4s). The distribution shapes are similar between groups as shown by a non-significant chi-square (df 4) = 1.2, p = 0.88).

The distribution of abnormal NCS-measures within subjects, both for the PNP27s (Fig. 1) and the CTS4s (Fig. 2), were similar within RYGB and community-control groups. Abnormality rates for different PNP27s and CTS4s cut-offs (Table 2) were similar in both groups with overlapping CIs, although slightly higher in RYGB patients for PNP27s cutoff 4 (18 % vs 13 %) and cutoff 5 (13 % vs 10 %) and CTS4s cutoff 4 (8 % vs 6 %) (Table 2).

**Table 2:** Abnormality rates for operational PNP and CTS diagnostics by PNP27s, CTS4s and CTS8s sumscores for different cut-offs (percentage of subjects with abnormal single NCS-variable counts greater than or equal to cut-off).

**Cutoff**	**RYGB group (n = 175)**	**Community-controls (n = 86)**
	**Abnormality-% (95 % CI)**	**Abnormality-% (95 % CI)**
PNP27s; range 0–27, and percentage ≥ cutoff		
4	18 (12,23)	13 (6,20)
5	13 (8,17)	10 (4,17)
6	9 (4,13)	8 (2,14)
7	5 (1,8)	6 (1,11)
CTS4s; range 0–4, and percentage ≥ cutoff		
2	15 (10,21)	15 (8,23)
3	13 (8,17)	12 (5,18)
4	8 (4,12)	6 (1,11)
CTS8s; range 0–8, and percentage ≥ cutoff		
3	21 (15,27)	na
4	16 (11,21)	na
5	13 (8,17)	na

RYGB: Roux-en-Y gastric bypass. PNP27s: Number of abnormal polyneuropathy-relevant NCS-measures. CTS4s: Number of abnormal carpal tunnel standard NCS-measures. CTS8s: Number of abnormal carpal tunnel standard plus extended NCS-measures defined in Table S2. na: not available. CI: confidence interval. Statistical comparison (chi-square test): All were non-significant (p > 0.30).

PNP27s and CTS4s sum scores and percentage scores were similar between groups (Table 3). No significant differences were found either for NCS-scores from arm and leg or from motor and sensory partial summations (Table 3). Mean abnormality scores were low, and the highest sub-scores were seen for leg- and sensory measures (Table 3). The highest abnormality percentage values for PNP were seen for sensory nerves (9.3 %, sd 11.5 %) and for leg nerves (7.8 %, sd 11.4 %). Mean abnormality percentage were larger for CTS4s (RYGB: 16 %, sd 31 %; Control: 15 %, sd 29 %) than for PNP27s (RYGB: 7 %, sd 8 %; Control: 6 %, sd 9 %). Median abnormality rates were also low in both groups (Table 3). In the BMI- and sex-matched sensitivity analysis, all PNP sum variables and the CTS4 sum variables in Table 3 were also not significant (Mann-Whitney p- values > 0.21).

**Table 3:** Sum and percent abnormal NCS measures for PNP- and CTS- sum-scores within 175 RYGB patients and 86 community-control subjects.

**Variable (number of measures)**^a^	**Sum-scores**		**Percent**^b^	
	**RYGB mean (sd)**	**Controls mean (sd)**	**RYGB median (90 % perc)**	**Controls median (90 % perc)**
CTS4s (4)	0.7 (1.2)	0.6 (1.2)	0 (75)	0 (75)
PNP27s (27)	1.9 (2.2)	1.7 (2.4)	4 (19)	4 (17)
PNP-arm (13)	0.8 (1.3)	0.6 (1.1)	0 (7)	0 (7)
PNP-leg (14)	1.1 (1.6)	1.1 (1.6)	0 (21)	7 (18)
PNP-sensory (12)	1.1 (1.4)	1.0 (1.6)	8 (25)	8 (25)
PNP-motor (15)	0.8 (1.2)	0.7 (1.3)	0 (17)	0 (13)

CTS: four standard carpal tunnel specific NCS measures (Table S2). PNP: polyneuropathy-relevant NCS measures; all 27 measures and motor/sensory and arm/leg subgroup were summed (Table S1).

Statistical comparison (Mann-Whitney *U* test) for group differences: All were non-significant (p > 0.28).

a Abnormal single NCS-measures (latency, conduction velocity or amplitude) outside age- and height-corrected laboratory-normal limits was coded as 1 and normal results as 0 before summation.

b Percent is sum-score divided by number of measures.

### 3.3 Clinical neurophysiologist’s diagnosis

PNP-ncs was a bit more prevalent in the RYGB-group (12 %) than the community-control-group (8 %); but CIs overlapped, and the difference was not significant (Table 4). Demyelinating PNP was not found. CTS-ncs was more prevalent in the RYGB group than in the community-control group (Table 4; 21 % vs 10 %, p = 0.04). Only two other entrapments (both ulnar elbow) were found in each group. Axonal mononeuropathy was found in 6 % of RYGB and 2 % of community-control groups, mainly caused by low peroneal motor or sensory amplitude or a low sensory ulnar nerve amplitude (Table 4).

**Table 4:** Final NCS-based neuropathy diagnosis by certified Clinical Neurophysiology MD specialists (recorded from the final EPJ-reports) in RYGB patients and community-controls.

	**RYGB-group (N = 175)**	**Community-controls (n = 86)**	**Chi-square**
	**Prevalence % (95 % CI)**	**Prevalence % (95 % CI)**	
PNP-ncs	12 (7,17)	8 (2,14)	0.9
CTS-ncs	21 (15,27)	10 (4,17)	4.1^1^
Ulnar entrapment	1 (0,3)	2 (0,6)	0.5
Mononeuropathy	6 (3,10)	2 (0,6)	1.9

EPJ: electronic patient journal. PNP-ncs: NCS-diagnosed polyneuropathy. CTS-ncs: NCS-diagnosed carpal tunnel syndrome. NCS: nerve conduction study. CI: confidence interval.

Statistical comparison (chi-square statistics with 1 degree of freedom): ^1^p = 0.04. Non-significant comparisons: p > 0.16.

Fourteen RYGB subjects (7 %) had diabetes at follow-up, and only one RYGB patient with present diabetes and four patients with preoperative diabetes had PNP-ncs. PNP-ncs was also unrelated to alcohol consumption habits and hypertension in RYGB patients. Four of 11 RYGB patients with a cancer history had PNP-ncs, as compared to 17 of 162 without (chi-square df1 = 6.46, p = 0.01).

CTS-ncs was found in 6 RYGB patients with diabetes (43 %) compared to 30 (19 %) of nondiabetic patients (p = 0.03). CTS-ncs was also unrelated to alcohol consumption habits, hypertension, and cancer-history in RYGB patients.

### 3.4 NCS-abnormality and BMI

RYGB patients were categorized into subgroups: lean (BMI < 25, n = 14), overweight (BMI 25–34.9; N = 82, and severely obese (BMI ≥ 35 N = 79). Axonal PNP-ncs prevalences were 21 %, 9 % and 14 % respectively (chi-square df2 = 2.38, p = 0.30). PNP27s score did not correlate with BMI (rho = 0.12, p = 0.12) in patients, while a positive association was found in controls (rho = 0.33, p = 0.002). The corresponding BMI-group CTS-ncs prevalences were 14 %, 16 % and 27 %. Although this trend was not significant (chi-square df2 = 3.2, p = 0.20; Cochran’s test for linear trend, p = 0.09), a weak but significant correlation between BMI and CTS4s (Spearman rho = 0.19, p = 0.01) was observed. CTS4s did also correlate with BMI in controls (Spearman rho = 0.43, p < 0.001).

### 3.5 Abnormality rates for single NCS-measures

NCS-variables used in abnormality indices Sum-PNP, CTS4s, in extended CTS measures, and for ulnar and peroneal entrapment, are shown in Supplementary Tables S1 and S2. Ten of 27 abnormality rates were larger for controls, three of ten being statistically significant, while 14 abnormality rates were larger for RYGB patients, five of 14 being statistically significant (Table S1).

High abnormality rates for single PNP variables were found for several sensory amplitudes. F-responses and other MCV and SCV measures were seldom abnormal (Table S1). Community-control group rates for single variable abnormality were often of equal or even higher magnitude particularly for foot SCV (Table S1).

High abnormality rates above 15 % in patients were found for CTS-specific measures like distal latencies of the median nerve, median SCV and the median-ulnar latency differences. However, the rates in community-controls were not different from those found in patients (Table S2).

## 4 Discussion

### 4.1 Polyneuropathy

The prevalence of PNP in the elderly population is about 7–8 % (Suzuki, 2013) although estimates from the US may be as high as 15 % in those over 40 years, possibly driven by obesity and diabetes as independent risk factors (Callaghan et al., 2020). This corresponds well with our findings (12 % in the overweight RYGB group and 8 % among the elderly community subjects), suggesting that bariatric surgery may not be an independent risk factor for PNP, provided adequate nutrition and use of supplements as described in the Nordic guidelines (Sandvik et al., 2018). A recent *meta*-analysis, restricted to maximum 12 months follow-up time, also concluded that bariatric surgery (either gastric sleeve or RYGB) may have a positive effect on symptoms and disability measured by questionnaires: Neuropathic Symptoms Score-NSS and Neuropathic Disability Scale-NDS (Aghili et al., 2019). A sensory PNP has been associated with RYGB previously, but its occurrence seems to be reduced in recent years (Thaisetthawatkul et al., 2010). A small observed difference between the two groups may equally well be caused by minor methodological factors and by factors related to overweight. Also, prophylactic treatment by Nordic guidelines have certainly contributed to overall rather low risk for neurological complication after bariatric surgery (Sandvik et al., 2018).

### 4.2 Carpal tunnel syndrome

The prevalence of clinically meaningful CTS is about 2 % for men and 3–5 % for women with highest occurrence in women above 65 years (Padua et al., 2016, Todnem and Sand, 2013). However, NCS-diagnosed CTS has been found in 9 % within a Dutch population (de Krom et al., 1992). Community dwellers in our control group had a comparable low prevalence of CTS-ncs, while the prevalence of CTS-ncs was higher than expected in the RYGB group. Obesity and diabetes are two of several well-known risk factors for CTS (Nathan et al., 1992, Geoghegan et al., 2004, Padua et al., 2016), although not always discussed in comprehensive reviews (O’Brien et al., 2017), and the risk seems to be greater for obesity than overweight (Lampainen et al., 2022). This trend was observed in our study too, although it did only reach significance in the CTS sum-score correlation analysis.

The rather high CTS-ncs rate may partly be explained as subclinical abnormal NCS results in some of the RYGB-group patients because we used extended diagnostic criteria comparing median and ulnar motor latencies to intrinsic hand muscles and comparing median and ulnar sensory velocities from the ring finger only in the RYGB-group. Additional measures reflecting both motor and sensory median-ulnar comparisons have been used and recommended for a long time (Uncini et al., 1993, Walters and Murray, 2001, Chang et al., 2006, Todnem and Sand, 2013), although their actual use in routine clinical neurophysiology is uncertain. It may be useful to also add median-radial conduction difference measures (Lee et al., 2011) and *trans*-carpal conduction measures (Chang et al., 2006) to future extended CTS-sum-scores. Although subclinical affection was also common in the community-control group, the almost identical mean values for the CTS sum, based on four standard segments in Table 3, suggest that the long-term risk difference for median neuropathy within the carpal tunnel after RYGB may be marginal.

In addition, CTS-prevalences in the literature are not easily comparable because the diagnostic criteria for CTS vary between countries, centres, and studies (Padua et al., 1999, Graham et al., 2006). The recommended *combined* clinical + NCS diagnosis is not always used, unless a confirmation before surgery is required (Middleton and Anakwe, 2014, Todnem and Sand, 2013). Diabetes, but not obesity, is associated with worse outcome after surgery for CTS (Zimmerman et al., 2017). The association between diabetes and CTS-ncs was also confirmed in our study.

### 4.3 NCS abnormality-scores for polyneuropathy and carpal tunnel syndrome

Operational PNP-diagnostics have been applied in several published guidelines for chronic inflammatory demyelinating polyneuropathy (CIDP) (van der Bergh et al., 2021). Similar international guidelines do not exist for axonal polyneuropathy, but guidelines from the ESTEEM project (Tankisi et al., 2005), and a recent review with suggested diagnostic algorithms for axonal PNP (Tankisi et al., 2020), have been published. Inter-physician differences in diagnosing PNP by NCS seem to be low in Europe (Tankisi et al., 2003).

Z-compounds are averaged Z-scores from selected nerves and have been used quite extensively to characterize axonal and diabetic PNP severity (Solders et al., 1993, Dyck et al., 2011a, Dyck et al., 2011b, Dunker et al., 2022). The presently defined simple categorical scores PNP27s, CTS4s and CTS8s were chosen to mirror parts the diagnostic process during electrodiagnostic evaluations; evaluations that depend on pattern recognition and the number of abnormal NCS-measures.

By comparing PNP-ncs prevalences in Table 4 with abnormality rates related to sum-score cut-offs in Table 2, it can be hypothesised that five or more abnormal NCS-measures, in addition to eventual entrapment-measure abnormalities, i.e. PNP27s ≥5, are required for a CN-based PNP-diagnosis. Abnormality in either three (Hughes et al., 2001) or two nerves (Van den Bergh and Piéret, 2004) has been suggested in guidelines for demyelinating PNPs, while abnormality in at least two nerves was deemed sufficient for axonal PNP (Tankisi et al., 2005). For operational CTS-diagnostics either four (CTS4s) or preferably eight items for CTS8s defined in Table S2 can be used. The RYGB abnormality rates for CTS8s was 21 % for cutoff ≥3 (Table 2), identical to the 21 % CTS-ncs prevalence in Table 4. Further development of sum-scores, by abnormality counts per nerve, and further validation of the utility of NCS-scores for quantification of severity and diagnostic cutoffs, should be performed in future studies of patients with well-characterized clinical PNP.

### 4.4 Sensory symptoms and signs

Thirty-nine percent of RYGB patients and 14 % among community-controls reported neuropathic arm symptoms. However, while six RYGB patients had CTS surgery performed before the present study, only two had surgery during four years after NCS, suggesting that these symptoms probably often were mild and not typical for CTS. Only 6 % of patients and 4 % of controls had both symptoms and sign sum-scores equal or greater than 2 (Table 1).

NCS reflect function in large-diameter myelinated nerve fibres. However, leg pain was reported by 36 % of patients and 7 % of community-controls, and the high sensory symptom rate among patients suggest that small fibres may be affected. Several factors, including diabetes and BMI has been found to be associated with small fibre neuropathy (SFN) (Johnson et al., 2021), but only 3 of 19 small-fibre tests had good diagnostic accuracy for clinically defined PNP in obese subjects (Callaghan et al., 2018). A study from 2011 reported that SFN could occur after bariatric surgery (Philippi et al., 2011). On the other hand, corneal nerve fibre length, seemingly reduced in obesity, may improve 12 months after bariatric surgery (Iqbal et al., 2021). However, *objective* sensory findings were scarce in both the RYGB and community-control groups in our study, and even less prevalent in patients (Table 1). Thus, SFN seems unlikely to be the main aetiology behind the increased pain symptoms in patients, although objective evaluation with intraepidermal nerve fibre density (IENFD) measures and thermal thresholds, necessary for complete SFN evaluation (Sène, 2018), were unavailable. Although speculative, obesity is associated with chronic pain in general, low back pain, and lumbar disc and osteoarthritic hip joint disorders that also cause leg pain (Okifuji and Hare, 2015, Zhou et al., 2021). A history of sciatica was indeed reported by more RYGB-patients than controls in the present study.

### 4.5 Strengths and limitations

Some methodological factors should be discussed. The study used objective NCS compared to a large control database, and the CN-labs in the two hospitals have used the current comprehensive NCS protocol for more than 10 years. A community-control group was examined during the same time-period with identical methods for single nerves. The sample sizes yielded adequate statistical power for hypothesis testing, although prevalence estimate precision would improve with a larger control group.

The moderate age-differences should not affect results because we used age- and height-corrected Z-scores for the NCS-abnormality rates. In addition, sex is of minor influence for NCS variables when height and age is corrected for. Although BMI-differences might influence sensory amplitudes (Buschbacher, 1998) we observed no or small group differences, and results were not changed in a sensitivity analysis within BMI- and sex-compatible subgroups.

The clinical examination had to be performed by surgeons or general practitioners for logistic reasons among RYGB patients. Clinical sensory examinations were performed according to the TCNS protocol in both groups, but neither a complete TCNS consensus-classification (Dyck et al., 2011a) nor a PNP-classification by the criteria published by Tesfaye et al. (2010), could be performed. The slightly higher clinically definite PNP prevalence in the community-control group (Table 1) could reflect the added evaluation of patellar and ankle tendon reflexes. However, we did not aim to compare clinically defined PNP prevalences statistically in the present study. The slight age difference between RYGB and community-control groups might also explain a part of the slightly higher sensory test abnormality occurrence in RYGB patients.

The extended CTS-variables were not recorded in the community-control group. This may have caused a slight underestimation of CTS-ncs prevalence in community-controls as compared to patients, a notion supported by similar quantitative CTS measure abnormality rates and sum-scores in both groups. The non-blinded design may in theory cause bias and possible underestimation of CTS in controls, however, the 10 % CTS-ncs prevalence among community-controls (Table 4) is close to prevalences in published population studies. Hence, it seems reasonable to conclude the long-term CTS risk may be only slightly elevated in RYGB patients, partly related to overweight and diabetes.

### 4.6 Conclusion

The prevalence of PNP-ncs among overweight subjects 10–15 years after RYGB is largely comparable to the prevalence in the general population, suggesting that malnutrition-induced neuropathy probably has been avoided by the recommended Nordic diet, supplements, and medications. Several patients had NCS results typical for CTS, and doctors should be aware of CTS-symptoms that might need further evaluation for clinical relevance, comorbidity like diabetes and treatment in this patient group. The risk for other neuropathies seemed to be very low. The present PNP-ncs was mainly a lower extremity axonal sensory-dominant polyneuropathy apparently unrelated to diabetes, alcohol, hypertension, and overweight, probably a partly cancer-history-related PNP and otherwise largely age-related idiopathic PNP. Summary scores for PNP and CTS abnormality quantification can be useful in future treatment- and follow-up studies. Sum-scores can be supplemented by corresponding Z-compounds for severity-quantification in future studies. Further analysis on how PNP-occurrence and PNP-severity among RYGB patients relates to pre-diabetes, and metabolic syndrome with central obesity (Kazamel et al., 2021) is of interest for future studies.

## Funding

The study was supported by the Liaison Committee for Education, Research and Innovation in Central Norway and Norwegian University of Science and Technology (NTNU) Norway, and St. Olav’s Hospital, Trondheim University Hospital, Norway.

## Declaration of competing interest

The authors declare that they have no known competing financial interests or personal relationships that could have appeared to influence the work reported in this paper.
