# Supplementary material for: Neuropathy 10–15 years after Roux-en-Y gastric bypass for severe obesity: A community-controlled nerve conduction study
Source: Clin Neurophysiol Pract. 2024 Mar 27;9:130–7. doi: 10.1016/j.cnp.2024.03.002 (PMC11015066; doi:10.1016/j.cnp.2024.03.002)
Supplement: Supplementary data 1 [file mmc1.docx]

| **Supplemental Table S1. Twenty-seven NCS variables selected for polyneuropathy diagnosis in the RYGB and community-control groups. Age- and height-corrected abnormality rates.** | | |
| --- | --- | --- |
|  | **RYGB group (n=175) Abnormality-%^a^ (95% CI)** | **Community-controls (n=86) Abnormality-%^a^ (95% CI)** |
| **Motor nerve conduction** | | |
| Median wrist CMAP | 3 (1,6) | 8 (2,14) |
| Median MCV | 4 (1,7) | 6 (1,11) |
| Median F-wave | 3 (1,6) | 2 (0,6) |
| Ulnar distal DML | 3 (1,6) | 1 (0,3) |
| Ulnar wrist CMAP | 2 (0,4) | 7 (2,12)^1^ |
| Ulnar MCV arm | 3 (1,6) | 0 (0,5) |
| Ulnar F-wave | 3 (1,6) | 5 (0,9) |
| Tibial ankle DML | 6 (2,9) | 3 (0,7) |
| Tibial ankle CMAP | 9 (5,13) | 9 (3,15) |
| Tibial MCV | 3 (1,6) | 8 (2,14) |
| Tibial F-wave | 5 (1,8) | 0 (0,6)^1^ |
| Peroneal ankle DML | 5 (1,8) | 3 (0,7) |
| Peroneal ankle CMAP | 14 (9,19) | 5 (0,9)^1^ |
| Peroneal lower leg MCV | 7 (4,11) | 7 (2,13) |
| Peroneal F-wave | 6 (2,10) | 1 (0,4) |
| **Sensory nerve conduction** | | |
| Mixed median amplitude | 11 (7,16) | 3 (0,7)^1^ |
| Median digit 3 SNAP | 18 (12,23) | 5 (0,9)^2^ |
| Ulnar digit 5 SNAP | 13 (8,17) | 8 (2,14) |
| Radial SNAP | 9 (5,13) | 2 (0,6)^1^ |
| Ulnar digit 5 SCV | 2 (0,4) | 2 (0,6) |
| Radial SCV | 5 (2,8) | 12 (5,18) |
| Sural SNAP | 9 (4,13) | 8 (2,14) |
| Peroneal SNAP | 17 (11,22) | 9 (3,15) |
| Medial plantar SNAP | 21 (15,27) | 23 (14,32) |
| Sural SCV | 2 (0,5) | 7 (2,13) |
| Peroneal SCV | 4 (1,7) | 12 (5,19)^1^ |
| Medial plantar SCV | 3 (0,5) | 16 (7,24)^1^ |
| ^a^Abnormality rates in percent exceeding 2sd from age and height corrected normal values.  CI: confidence interval, DML: distal motor latency, MCV: motor conduction velocity, CMAP: compound motor action potential amplitude, SCV: sensory conduction velocity, SNAP: sensory nerve action potential amplitude, F-wave: Minimal F-latency minus M-latency.  Descriptive statistical comparison for single NCS-measure abnormality scores (chi-square test): ^1^p<0.05, ^2^p<0.005. | | |
