# Supplementary material for: Neuropathy 10–15 years after Roux-en-Y gastric bypass for severe obesity: A community-controlled nerve conduction study
Source: Clin Neurophysiol Pract. 2024 Mar 27;9:130–7. doi: 10.1016/j.cnp.2024.03.002 (PMC11015066; doi:10.1016/j.cnp.2024.03.002)
Supplement: Supplementary data 2 [file mmc2.docx]

| **Supplemental Table S2. NCS variables selected for entrapment diagnosis in the RYGB and community-control groups. Age and height corrected abnormality rates.** | | |
| --- | --- | --- |
|  | **RYGB group (n=175) Abnormality-%^a^ (95% CI)** | **Community-controls (n=86) Abnormality-%^a^ (95% CI)** |
| **Median nerve standard measures** |  |  |
| Median thenar DML | 18 (12,23) | 15 (8,23) |
| Mixed median volar CV | 17 (11,22) | 15 (8,23) |
| Median digit 3 SCV | 15 (10,21) | 13 (6,20) |
| Digit 5-3 SCV difference | 16 (11,22) | 15 (8,23) |
| **Median nerve extended measures** | | |
| Lumbrical-interossal DML difference | 16 (10,21) | na |
| Median lumbrical DML | 20 (14,26) | na |
| Digit 4 median-ulnar SCV difference | 14 (9,19) | na |
| Median dig 4 SCV | 25 (19,32) | na |
| **Ulnar nerve** |  |  |
| Ulnar CMAP evoked above sulcus | 4 (1,7) | 7 (2,12) |
| Ulnar MCV across sulcus (10 cm) | 6 (2,9) | 7 (2,12) |
| **Peroneal nerve** |  |  |
| Peroneal CMAP evoked from popliteal fossa | 18 (12,23) | 3 (0,7)^2^ |
| Peroneal MCV across fibular head (10 cm) | 4 (1,7) | 5 (0,9) |
|  | | |

^a^Abnormality in percent exceeding 2sd from age and height corrected normal values.

CI: confidence interval, DML: distal motor latency, MCV: motor conduction velocity, CMAP: compound motor action potential amplitude, SCV: sensory conduction velocity. na: Measures not acquired in the community-control group.

Descriptive statistical comparison for single NCS-measure abnormality scores (chi-square test): ^2^p<0.005.
